# Dual Blockade of TNF and IL-17A Inhibits Inflammation and Structural Damage in a Rat Model of Spondyloarthritis

**DOI:** 10.3390/ijms23020859

**Published:** 2022-01-13

**Authors:** Ihsan Hammoura, Renee H. Fiechter, Shaughn H. Bryant, Susan Westmoreland, Gillian Kingsbury, Wendy Waegell, Sander W. Tas, Dominique L. Baeten, Marleen G. H. van de Sande, Melissa N. van Tok, Leonie M. van Duivenvoorde

**Affiliations:** 1 Amsterdam Rheumatology and Immunology Center, Department of Clinical Immunology and Rheumatology, Amsterdam University Medical Centers, University of Amsterdam, 1105 AZ Amsterdam, The Netherlands; i.hammoura@amsterdamumc.nl (I.H.); r.h.fiechter@amsterdamumc.nl (R.H.F.); s.w.tas@amsterdamumc.nl (S.W.T.); dominique.baeten@ucb.com (D.L.B.); melissavantok@gmail.com (M.N.v.T.); l.m.vanduivenvoorde@amsterdamumc.nl (L.M.v.D.); 2 Department of Experimental Immunology, Amsterdam University Medical Centers, University of Amsterdam, 1105 AZ Amsterdam, The Netherlands; 3 AbbVie Bioresearch Center, Worcester, MA 01605, USA; shaughn.bryant@abbvie.com (S.H.B.); susan.westmoreland@abbvie.com (S.W.); gill@manaTbio.com (G.K.); wendy.waegell@abbvie.com (W.W.)

**Keywords:** spondyloarthritis, anti-TNF treatment, anti-IL 17A treatment, dual blockade, new bone formation, animal disease model

## Abstract

The tumor necrosis factor (TNF) and IL-23/IL-17 axes are the main therapeutic targets in spondyloarthritis. Despite the clinical efficacy of blocking either pathway, monotherapy does not induce remission in all patients and its effect on new bone formation remains unclear. We aimed to study the effect of TNF and IL-17A dual inhibition on clinical disease and structural damage using the HLA-B27/human β2-microglobulin transgenic rat model of SpA. Immunized rats were randomized according to arthritis severity, 1 week after arthritis incidence reached 50%, to be treated twice weekly for a period of 5 weeks with either a dual blockade therapy of an anti-TNF antibody and an anti-IL-17A antibody, a single therapy of either antibody, or PBS as vehicle control. Treatment-blinded observers assessed inflammation and structural damage clinically, histologically and by micro-CT imaging. Both single therapies as well as TNF and IL-17A dual blockade therapy reduced clinical spondylitis and peripheral arthritis effectively and similarly. Clinical improvement was confirmed for all treatments by a reduction of histological inflammation and pannus formation (*p* < 0.05) at the caudal spine. All treatments showed an improvement of structural changes at the axial and peripheral joints on micro-CT imaging, with a significant decrease for roughness (*p* < 0.05), which reflects both erosion and new bone formation, at the level of the caudal spine. The effect of dual blockade therapy on new bone formation was more prominent at the axial than the peripheral level. Collectively, our study showed that dual blockade therapy significantly reduces inflammation and structural changes, including new bone formation. However, we could not confirm a more pronounced effect of dual inhibition compared to single inhibition.

## 1. Introduction

Spondyloarthritis (SpA) is the second most common form of chronic inflammatory arthritis [1]. SpA can be divided into two subcategories according to its clinical joint manifestations: axial SpA, including radiographic (or ankylosing spondylitis) and non-radiographic axial SpA, and peripheral SpA, including psoriatic arthritis (PsA). Also, various extra-articular manifestations such as inflammatory bowel disease, uveitis or psoriasis can occur [1]. Besides inflammation, the joints of SpA patients can be affected by bone remodeling, including pathological new bone formation which leads to ankylosis and often disability [2]. Although the aetiology remains unknown, the tumor necrosis factor (TNF) and interleukin-23/-17 (IL-23/IL-17) pathways dictate SpA pathogenesis [1,3,4,5,6,7,8,9,10]. Blockade of either pathway clearly inhibits inflammation and improves key clinical outcomes, such as the Assessments of SpondyloArthritis International Society response (ASAS) criteria and patient-reported outcomes [7,10,11,12,13,14,15,16]. However, biological treatments that block one of these pathways are only effective in up to 70% of SpA patients and induce remission in fewer patients [7,10,11,12,13,14,15,16,17,18]. Although some promising results imply that the IL-17A blockade might reduce new bone formation [19,20,21,22], the optimal strategy to halt this pathological process remains a clinical challenge.

Previous pre-clinical and clinical studies imply that the TNF and IL-23/IL-17 pathways operate independently from one another [7,23,24,25] and might contribute differentially to disease pathogenesis [19,20,21,26]. For instance, biological treatments targeting TNF seem effective in reducing synovial inflammation [27], while biological treatments against IL-17A seem more effective in ameliorating entheseal inflammation [28,29] and might target new bone formation as suggested in our previous preclinical studies [21]. Therefore, combined TNF and IL-17A blockade could potentially synergistically target both inflammation and new bone formation. Dual blockade of TNF and IL-17A using the combination of soluble IL-17 receptor (sIL-17R) and TNF binding protein (TNFBP) [30] or combined single/dual variable domain bispecific neutralizing antibodies [31] was shown to be effective in the human TNF transgenic mouse model of arthritis and the collagen-induced arthritis model (i.e., preclinical models of rheumatoid arthritis (RA)). Safety was also shown in clinical trials using TNF and IL-17A-targeted dual variable domain immunoglobulin (ABT122) in RA [32] or PsA patients [33]. A modest increase in efficacy was seen in other RA clinical trials using combined single neutralizing antibodies [34]. However, appropriate efficacy studies in axial and peripheral SpA are lacking.

We hypothesized that the dual blockade of TNF and IL-17A effectively targets both inflammation and new bone formation in SpA. To test this hypothesis we performed dual blockade of TNF and IL-17A in the *Mycobacterium tuberculosis* (*M. tub.*) induced HLA-B27/Human βeta 2 microglobulin (Huβ2m) rat model of SpA [35].

## 2. Results

### 2.1. Dual Tumor Necrosis Factor (TNF) and Interleukin-17A (IL-17A) Blockade Reduces Clinical Spondylitis and Peripheral Arthritis

**Figure 1:**
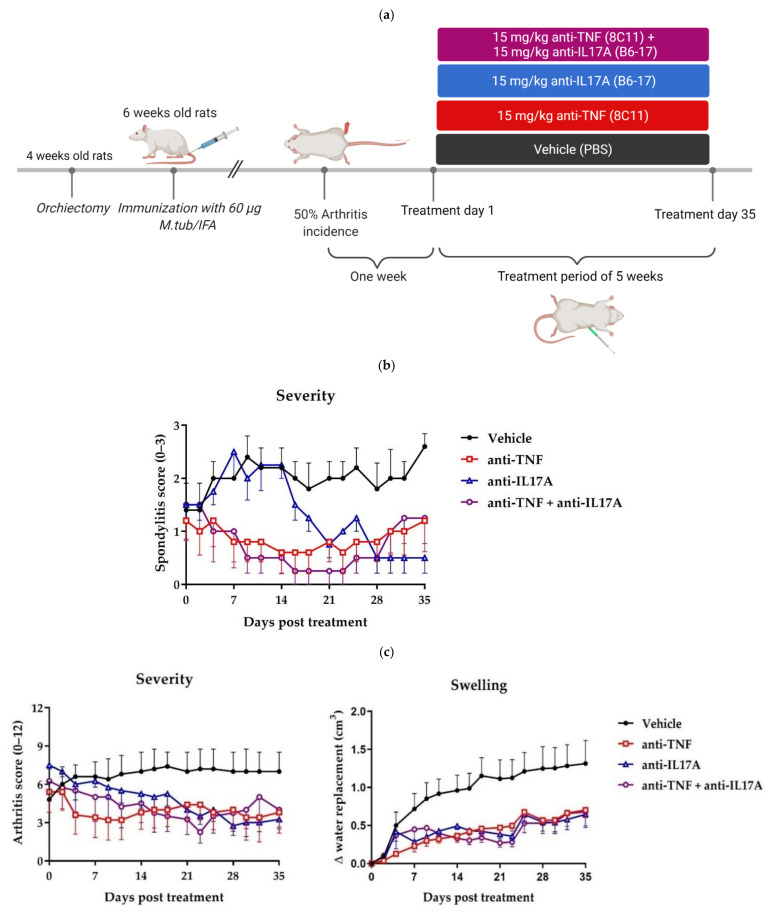
Tumor necrosis factor (TNF) and interleukin-17A (IL-17A) dual blockade therapy reduces axial and peripheral clinical disease. (**a**) Study design. Male HLA-B27/huβ2m transgenic rats were orchiectomized at four weeks of age and immunized with 60 µg *Mycobacterium tuberculosis* (*M. tub.*) at six weeks of age. One week after arthritis incidence reached 50%, the rats were randomized according to arthritis severity to be treated twice weekly with intraperitoneal injections with either 15 mg/kg anti-TNF and 15 mg/kg anti-IL-17A (8C11 and B6-17, *n* = 4), 15 mg/kg anti-TNF (8C11, *n* = 5), 15 mg/kg anti-IL-17A (B6-17, *n* = 4), or PBS as vehicle control (*n* = 5) for five weeks. An experimentally blinded observer scored spondylitis severity (0–3 per tail), and peripheral arthritis severity (0–3 per paw) and hind paw swelling (in cm^3^ water replacement using plethysmography) at multiple time points. (**b**) Spondylitis severity. A significant decrease was seen for spondylitis score for dual blockade therapy (*p* = 0.031) and anti-TNF (*p* = 0.031) versus vehicle; a trend was seen for anti-IL-17A versus vehicle (*p* = 0.165). (**c**) Peripheral arthritis severity and hind paw swelling. Numerical, but non-significant, decreases were seen for the combinational and both single therapy groups compared to the vehicle. Hind paw swelling was significantly decreased for combinational (*p* = 0.008), anti-TNF (*p* = 0.006) and anti-IL-17A (*p* = 0.011) treatments compared to vehicle control. Figure (**a**) was created with BioRender.com.

We assessed the effects of 5 weeks of dual blockade of TNF and IL-17A, TNF inhibitor monotherapy, and IL-17A inhibitor monotherapy, compared to vehicle control, on the severity and the extent of spondyloarthritis manifestations in the *M. tub.*-induced HLA-B27/huB2m tg rats (Figure 1a). All treatment regimens showed a decrease in spondylitis severity compared to the vehicle treated group, which reached significance for the combination therapy (*p* = 0.031) and anti-TNF monotherapy groups (*p* = 0.031), while a numerical decrease was observed in the anti-IL17A monotherapy group without statistical significance (*p* = 0.164) (Figure 1b). The clinical severity scores for peripheral arthritis decreased over time in all treatment groups compared to the vehicle treated group, but did not reach significance. Swelling of the hind paws indicative of arthritis decreased significantly compared to the vehicle treated group for the anti-TNF monotherapy (*p* = 0.006), anti-IL-17A monotherapy (*p* = 0.011) and the dual blockade therapy groups (*p* = 0.008) (Figure 1c). These data indicate that TNF and IL-17A dual blockade effectively reduced clinical spondylitis and peripheral arthritis.

### 2.2. TNF and IL-17A Dual Blockade Therapy Reduces Inflammation and New Bone Formation in the Axial and Peripheral Joints

**Figure 2:**
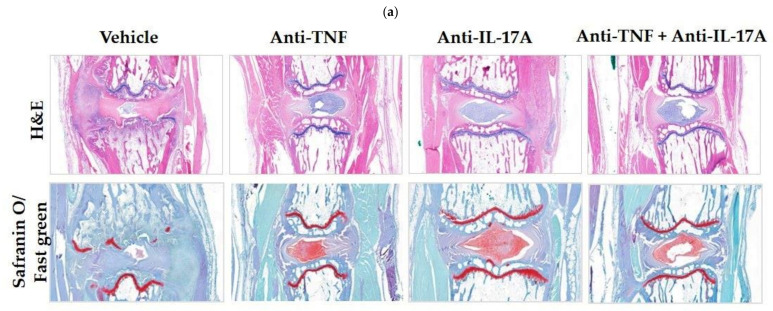

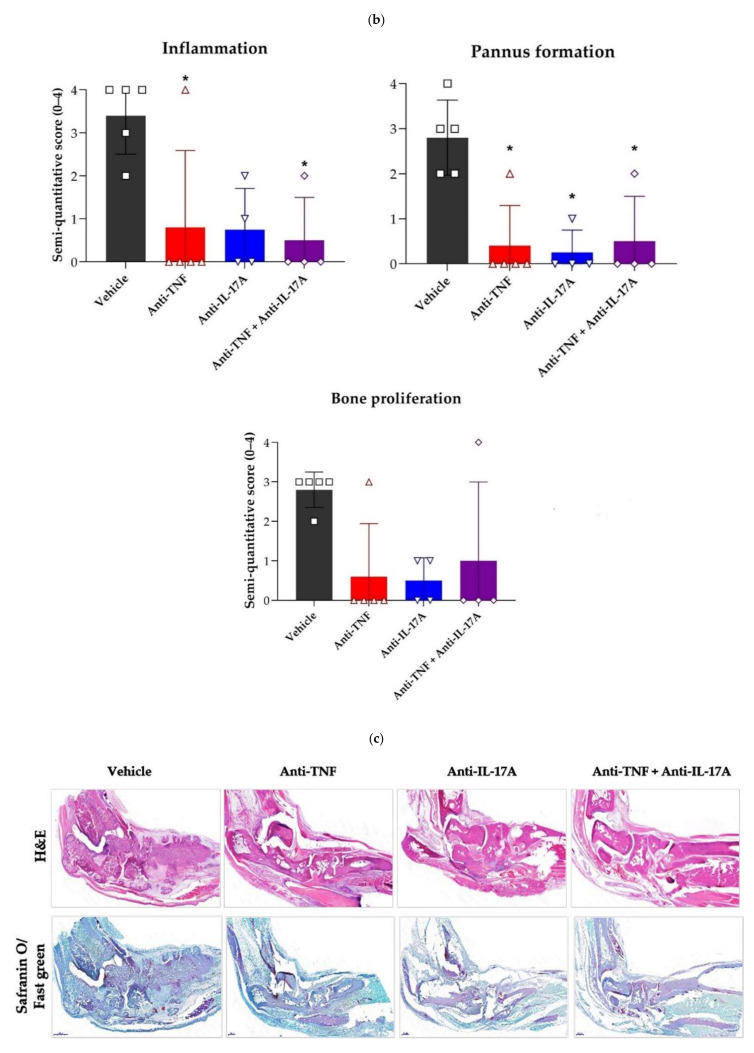

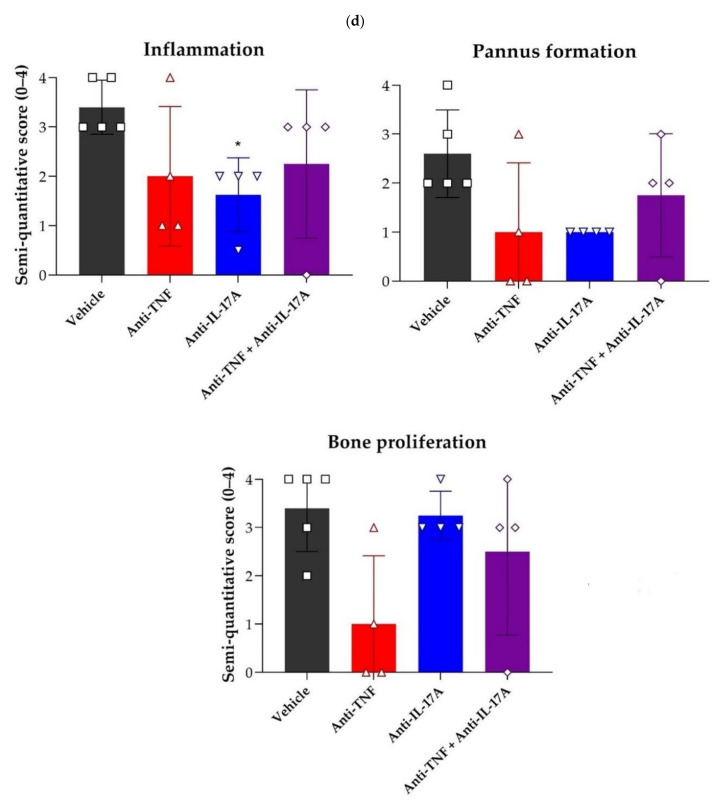
TNF and IL-17A dual blockade therapy reduces inflammation and bone proliferation in the axial and peripheral joints. (**a**) Representative caudal spine sections stained for hematoxylin and eosin (H&E) for rats who were treated with anti-TNF and anti-IL-17A (8C11 and B6-17, *n* = 4), anti-TNF (8C11, *n* = 4–5), anti-IL-17A (B6-17, *n* = 4), or vehicle control (*n* = 5). (**b**) Experimentally blinded semi-quantitative scoring (0–4) of inflammation, pannus formation and bone proliferation of histologically stained caudal spine sections showed significant decreases of inflammation for dual blockade (*p* = 0.036) and anti-TNF (*p* = 0.039). Pannus formation was significantly reduced for all treatments compared to vehicle control (anti-TNF, *p* = 0.016; anti-IL17A, *p* = 0.021; dual blockade, *p* = 0.037). A numerical decrease of bone proliferation was seen for all treatment modalities compared to vehicle control. (**c**) Representative ankle sections stained for H&E for the three different treatment modalities and vehicle control. (**d**) Experimentally blinded semi-quantitative scoring (0–4) of histologically stained ankle sections showed a significant decrease for inflammation for anti-IL17A (*p* = 0.047) compared to vehicle control. *, *p* ≤ 0.05.

To substantiate the clinical data, we evaluated caudal spine and ankle sections histologically stained for hematoxylin/eosin and Safranin O/Fast Green (Figure 2a,c). At the level of the caudal spine, significant reduction of inflammation was shown for dual blockade (*p* = 0.036), and anti-TNF (*p* = 0.039) therapies compared with the vehicle control (Figure 2b). Similarly, significant reduction of pannus formation was shown for dual blockade (*p* = 0.037), anti-TNF (*p* = 0.016) and anti-IL17A (*p* = 0.021) therapies compared with the vehicle control. Compared with vehicle control, a numerical decrease of bone proliferation in the spine was seen for all treatment modalities.

At the level of the ankle, histological analysis also confirmed a significant reduction of inflammation for the anti-IL17A therapy (*p* = 0.047) compared to the vehicle control, while dual blockade and anti-TNF therapies only showed a trend of reduced inflammation (Figure 2d), which did not reach significance. All treatments regimes showed a numerical decrease of pannus formation and bone proliferation in the ankle joints compared to the vehicle control (Figure 2d). Together, these data further confirm the clinical effectiveness of the dual blockade therapy in reducing inflammation and new bone formation at the level of the axial and peripheral joints on both the subclinical and clinical levels, with greater efficacy seen at the axial joints.

### 2.3. Micro-Computed Tomography (CT) Imaging Further Confirms the Efficacy of TNF and IL-17A Dual Blockade Therapy in Reducing Structural Damage in Axial and Peripheral Disease

**Figure 3:**
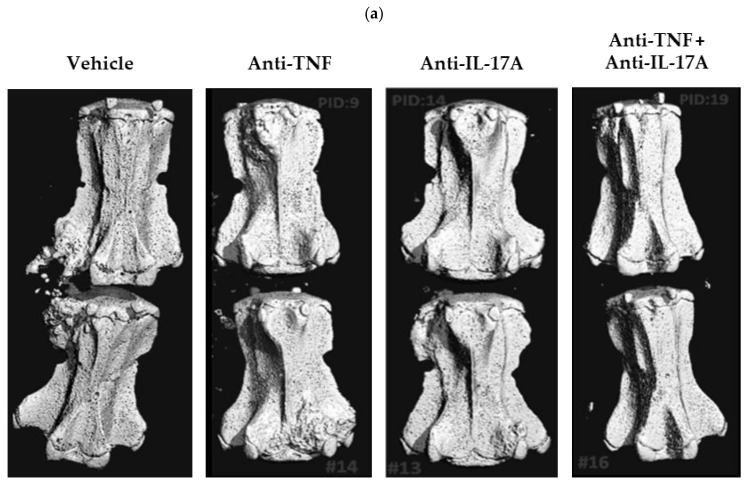

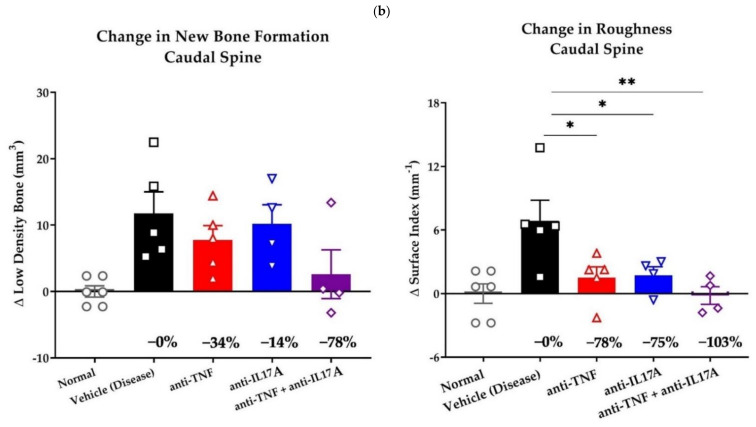
TNF and IL-17A dual blockade therapy affects bone remodeling in axial joints. (**a**) Representative micro-computed tomography (CT) image of the Caudal 3-Caudal 4 (Ca3-Ca4) vertebrae of HLA-B27/huβ2m transgenic rats who were treated five weeks with anti-TNF and anti-IL-17A (8C11 and B6-17, *n* = 4), anti-TNF (8C11, *n* = 5), anti-IL-17A (B6-17, *n* = 4), or vehicle control (*n* = 5). (**b**) Scoring of micro-CT images of Ca3-Ca4 vertebrae of age and gender matched non-diseased rats and the three treatment modalities compared to vehicle control. Compared to vehicle control, all three treatment modalities showed numerical, but non-significant, decreases for new bone proliferation and a significant decreases for roughness (anti-TNF, *p* = 0.019; anti-IL17A, *p* = 0.036; dual blockade, *p* = 0.004). The dual blockade therapy group showed the strongest decreases (−78% for new bone formation and −103% for roughness). *, *p* ≤ 0.05; **, *p* ≤ 0.01.

**Figure 4:**
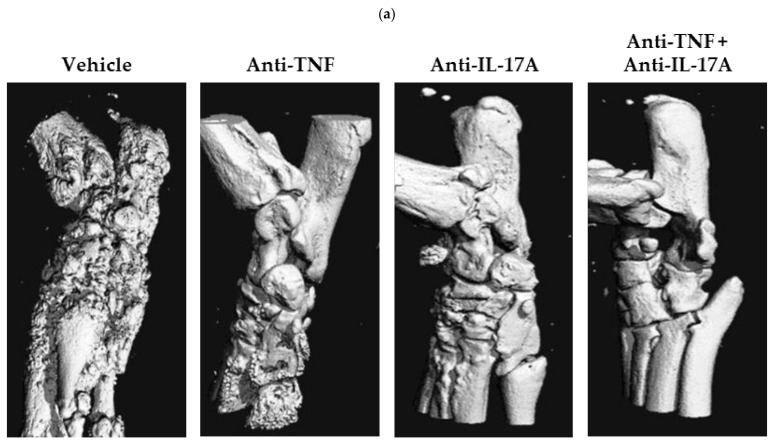

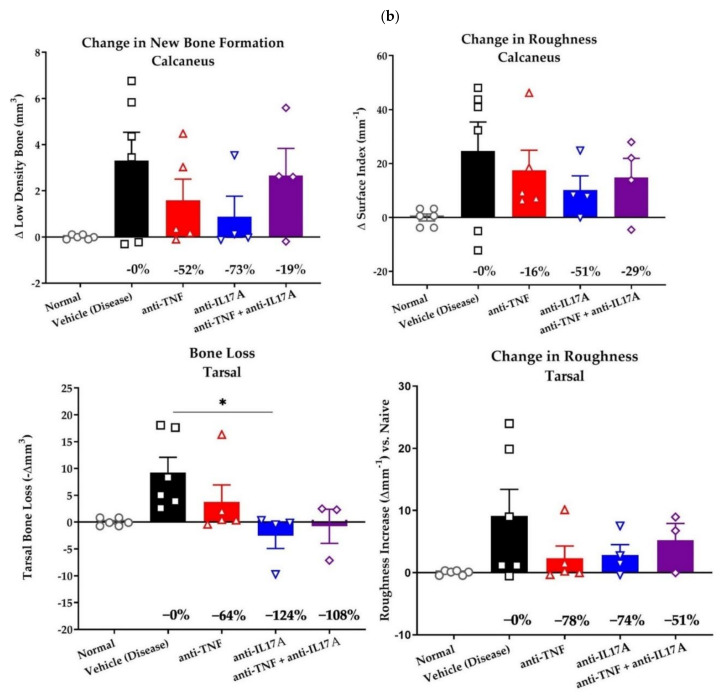
TNF and IL-17A dual blockade therapy affects bone remodeling in peripheral joints. (**a**) Representative micro-CT image of the ankles of HLA-B27/huβ2m transgenic rats who were treated with anti-TNF and anti-IL-17A (8C11 and B6-17, *n* = 3–4), anti-TNF (8C11, *n* = 5), anti-IL-17A (B6-17, *n* = 4), or PBS (*n* = 5). (**b**) Scoring of micro-CT images of calcaneus and tarsal bones of age and gender matched non-diseased rats and the three treatment modalities compared to vehicle control. Numerical, but non-significant, decreases were seen for the dual and both single treatment groups compared to vehicle control for the assessed regions, reaching significance for anti-IL-17A (*p* = 0.031) treatment on tarsal bone loss. *, *p* ≤ 0.05.

Next, we performed micro-CT imaging of the caudal spine of the anti-TNF, anti-IL-17A and dual blockade therapy-treated rats to evaluate the effects on structural damage in axial disease in comparison to vehicle treated and normal non-diseased rats. Micro-CT imaging revealed a marked reduction of new bone formation in the dual blockade treatment group compared with the vehicle control group (−78%), and a less pronounced reduction for anti-TNF (−34%) and anti-IL-17A (−14%) monotherapy, respectively, although none reached significance (Figure 3a,b). Similarly, bone roughness (depicting both erosions and new bone formation) was markedly and significantly reduced for the dual blockade therapy group compared with vehicle control (−103%, *p* = 0.004), and slightly less for anti-TNF (−75%, *p* = 0.019) and anti-IL-17A (−78%, *p* = 0.036) monotherapy, respectively (Figure 3a,b). To evaluate the effect of dual blockade therapy on peripheral disease, we assessed the calcaneus and tarsal bones using the same approach mentioned above. Micro-CT imaging revealed a significant reduction of bone loss at the level of the tarsals for anti-IL17A (−124%, *p* = 0.013) monotherapy compared to vehicle-treated, diseased rats (Figure 4b). A similar, albeit non-significant, reduction was observed for anti-TNF (−64%) and dual blockade (−108%) therapies (Figure 4b). Moreover, dual blockade therapy numerically decreased new bone formation (−19%) and roughness (−29%) at the calcaneus, and roughness (−51%) at the tarsal bones, though none were statistically significant (Figure 4a,b). These reductions were also seen for anti-TNF (−52%, −16%, and −78%, respectively) and anti-IL-17A monotherapies (−73%; −51%; and −74%, respectively) (Figure 4A,B). Together, these data further confirm that anti-TNF and anti-IL-17A dual blockade therapy decreases structural damage including new bone formation at the level of the axial and peripheral joints, but with potentially more effectiveness at level of the axial joints.

## 3. Discussion

The TNF and IL-23/IL-17 axes are major contributes to pathology in SpA [3,36]. Both of these pro-inflammatory pathways are considered important therapeutic targets in the treatment of axSpA. However, despite the clinical efficacy of biological disease-modifying anti-rheumatic drugs (DMARDS) blocking these pathways in reducing inflammation, their impact on new bone formation is not quite clear. Moreover, targeting new bone formation clinically remains a challenge. Thus, new therapeutic regimes and strategies are required. In this study, we hypothesized that the dual blockade of TNF and IL-17A would effectively reduce both inflammation and structural damage in the context of spondyloarthritis. To test this hypothesis, we utilized the *M. tub.* induced HLA-B27/Huβ2m tg rat SpA disease model which offers a great opportunity to study the effects of existing and new treatment regimens on both inflammation and bone remodeling. In this experimental disease model, we tested the effects of single or dual blockade of IL-17A and TNF on axial and peripheral disease in combination with an in-depth assessment of inflammation and structural damage at different disease sites.

In this study, TNF blockade resulted in a significant reduction in the severity of spondylitis associated with a significant reduction of axial inflammation and bone roughness at the level of the caudal spine. These results confirm the findings of the studies on the transmembrane TNF (tmTNF) transgenic mouse model of SpA, in which it has been shown that the overexpression of tmTNF drives osteoproliferative joint inflammation seen in SpA. TNF receptor I signaling is essential for inflammation and TNF receptor II signaling contributes to pathological new bone formation [37,38]. Moreover, anti-TNF treatment with the etanercept in the same mouse model resulted in a reduction in axial and peripheral inflammation and a reduced bone surface roughness at the level of the caudal spine [38]. In the clinic, TNF inhibitors are established in disease management of axSpA with efficacy in reducing inflammation [39,40,41]. Earlier clinical studies on TNF inhibitors failed to show a clear effect on radiographic progression [6,26,42]. However, reduction in structural alterations was observed with a longer follow up period and in prospective studies [43,44,45,46]. Thus, these observations highlight the efficacy of TNF inhibitors in the reduction of inflammation and link the TNF pathway to structural damage.

We have also demonstrated that treatment with anti-IL-17A monotherapy resulted in the significant reduction in severity of arthritis and axial inflammation. IL-17A blockade also resulted in a significant reduction of bone roughness at the level of the caudal spine and bone remodeling at the level of the tarsals. These results confirm previous studies in the HLA-B27/Huβ2m tg rat model where both preventive and therapeutic inhibition of IL-17A reduced inflammation and new bone formation [22]. Moreover, IL17A inhibitors are effective in the treatment of axSpA in the clinic [14,47,48,49]. IL-17A inhibitors effectively reduce axial inflammation as observed on MRI scans [50] and, clinical trials have shown that around 79% of axSpA patients receiving secukinumab show no radiographic progression which was sustained throughout 4 years of follow up [20]. Therefore, like TNF inhibitors, IL-17A inhibitors are considered effective in the management of axSpA.

Nevertheless, despite these positive observations neither TNF nor IL-17A inhibitors can normalize both inflammation and bone remodeling in all patients and complete remission with either therapeutic remains low. The latter further confirms the need for novel treatment strategies. In our study dual anti-TNF and anti-IL-17A therapy resulted in significant reduction of spondylitis and swelling at the level of the peripheral joints. This was also associated with a significant reduction of axial inflammation, bone roughness at the level of the caudal spine and bone loss at the level of the tarsals. Our results indicate that dual blockade of anti-TNF and anti-IL-17A is effective in experimental SpA and may have added clinical value. In agreement with our results, the dual blockade therapy of anti-IL17A and anti-TNF (infliximab) in the human TNF mouse model, resulted in a significant reduction of synovitis, synovial hyperplasia, and cartilage damage with a similar efficacy as high dose TNF inhibition. Dual inhibition also showed a superior effect on bone remodeling with a significant reduction of the bone remodeling markers RANKL and osteoprotegerin [31]. In clinical trials, dual blockade therapy is relatively safe in PsA and modestly effective in RA [32,33]. However, the efficacy of dual blockade on SpA patients has not been studied yet.

Dual blockade of TNF and IL-17A demonstrates efficacy in controlling inflammation and structural damage of the caudal spine with a smaller effect on the peripheral joints in the *M. tub.* induced HLA-B27/Huβ2m tg rat SpA disease model. In light of this data, we hypothesize that early, profound and sustained suppression of inflammation is essential to arrest or prevent structural damage. This hypothesis is consistent with previous observations in the non-immunized HLA-B27/Huβ2m tg rats where no new bone formation is observed in the absence of inflammation [51,52,53], and in the tmTNF tg mice where the prevention of inflammation via the knockout of TNFR1 also results in a reduction of new bone formation [37]. Follow-up studies in this rat model utilizing dual blockade therapy with various treatment regimens such as early versus late treatment, sustained versus intermittent, or sub-optimal versus optimal dosages of both inhibitors will help to elucidate a full understanding of the underlying pathological mechanisms, and help determine the ideal timing for therapeutic intervention to stop inflammation and prevent any subsequent structural damage. This in turn will likely also help to improve the quality of life of patients.

The main limitation of our study was the small group sizes, which probably accounted for the fact that we observed only numerical differences for several outcomes and could not perform formal statistical analysis comparing various treatment arms. Moreover, molecular data could have helped to elucidate the individual and synergistic effects of TNF and IL-17A inhibition, but this was outside the scope of this proof-of-concept study. Despite these limitations, our study is the first to demonstrate the positive effect of TNF and IL-17A dual blockade therapy in the *M. tub*. induced HLA-B27/Huβ2m tg rats.

In conclusion, our data indicate that treatment with a dual blockade with TNF inhibitor and IL-17A inhibitor impacts both axial and peripheral inflammation as well as structural damage. Further preclinical and clinical research is needed to assess whether deep and sustained control of inflammation is needed to fully abrogate structural damage in (preclinical) SpA.

## 4. Materials and Methods

### 4.1. Rats

The transgenic (tg) 21-3 (HLA-B27/Huβ2m) and 283-2 (Huβ2m) rat lines [35] of the Lewis background were bred and housed (four per cage) in individually ventilated cages at the animal research facility at the Amsterdam Medical Center (AMC). For the experiments, male F1 rats [21-3 × 283-2] were used to keep variation between animals as low as possible. The female rats from these litters were used in another experiment for ethical reasons. Animals received appropriate cage enrichment and ad libitum water and chow. The AMC Animal Ethics Committee approved all experiments.

### 4.2. Orchiectomy and Immunization

At four weeks of age, orchiectomy was performed using a standard protocol (described in Van Tok et al., 2017 [35]) to prevent epididymo-orchitis [52]. Following a wash-out period of two weeks, the rats were immunized with 60 µg of pulverized heat-inactivated *Mycobacterium tuberculosis* (*M. tub.*, Difco, Sparks, MD, USA) in 100 μL Freund’s incomplete adjuvant (IFA) (Chondrex, Woodinvill, WA, USA) [35] by an intradermal injection at the tail base under isoflurane anesthesia.

### 4.3. Clinical Scoring

The presence of arthritis in the paws was identified macroscopically, the severity was scored 0–3 per paw (0 = normal joints, 1 = one swollen joint, 2 = two or more swollen joints, and 3 = swelling of the entire paw and/or ankyloses), and a cumulative arthritis severity score for all four paws was calculated. Digital swelling (in cm^3^) was measured using plethysmometry and normalized to the day of therapeutic treatment initiation. Spondylitis, characterized by the presence of swelling and bumps in the tail, was identified macroscopically and scored 0–3 per tail. Clinical scoring and plethysmography were performed by an observer, blinded for the treatment. Humane endpoints were 20% body weight loss or the complete swelling of two paws.

### 4.4. Treatment

One week after the incidence of arthritis reached 50%, the rats were randomized according to arthritis scores into four groups of five rats and were treated for 5 weeks with twice weekly intraperitoneal injections with either 15 mg/kg of a rat-specific anti-IL-17A antibody (clone B6-17, AbbVie), 15 mg/kg a rat-specific anti-TNF antibody (clone 8C11, AbbVie), a combination of both, or PBS as a vehicle control. The dosages of both antibodies were determined by AbbVie through titration in a rat collagen-induced arthritis model for efficacy (data not shown). We chose to use the total dosage of both antibodies for the dual blockade treatment to see a clear effect.

The rats of two cages started treatment earlier as arthritis was developing more rapidly than in the other cages. Two rats, one from the anti-IL-17A and one from the dual blockade therapy group, developed only very mild arthritis which was not progressive during the experiment. Therefore, they were excluded and left out of further analyses.

### 4.5. Ex Vivo Micro-Computed Tomography (Micro-CT) Analysis

The lumbar spine, sacroiliac pelvis region, knees, left hind paw and tails of all animals were isolated, fixed with 4% formalin and transferred to 70% ethanol to be imaged by micro-CT. First, the vehicle-treated rats were scanned to detect areas with bone changes—including new bone formation, roughness and bone loss—subsequently, the affected areas were scanned for the rats of the single and dual blockade therapy groups. Approximately 1000 slices were included (18 mm) in the analysis of caudal spine, compromising 2 caudal vertebra (Ca3-Ca4) and the gap between them. The segmentation threshold used was 200 to 320 mg of HA/cm^3^ to include low density (partially mineralized) bone and exclude cortical bone; endosteal surface was masked. Moreover, in the analysis of distal calcaneus, 200 slices (3.6 mm) were utilized compromising the distal end of calcaneus with a segmentation threshold of 220–320 mg of HA/cm^3^ to estimate low-density new periosteal bone formation; endosteal surface was also masked. In the analysis of rear paw tarsals, 200 slices (3.6 mm) comprising the navicular bone and tarsals were used with a segmentation threshold 350 mg of HA/cm^3^ to include remaining high density cortical bone.

### 4.6. Histopathology

After micro-CT analysis, the hind paws and tails of the rats were decalcified with OsteoSoft decalcifier solution (no. 101728; Merck, Burlington, MA, USA) and embedded in paraffin. Five micrometer sections were stained for hematoxylin and eosin (H&E) or Safranin O Fast Green. For H&E staining, sections were incubated in Mayer’s hematoxylin (no. MHS16) and Eosin Y solution (no. HT110116) (both from Sigma, St. louis, MO, USA). For Safranin O Fast Green staining, sections were incubated in Weigert’s iron hematoxylin (no. HT1079), 0.1% fast green FCF (no. F7252), and 0.1% Safranin O solution (no. HT90432) (all from Sigma, St. louis, MO, USA). The sections were subsequently dehydrated and embedded in Entellan (no. 1.07961.0100; Merck, Burlington, MA, USA). Separate experimentally blinded observers scored the caudal spine, calcaneus, and tarsal regions semi-quantitatively (0–4) for inflammation, bone proliferation and pannus formation.

### 4.7. Statistical Analysis

The data were analyzed using GraphPad Prism 6 and IBM SPSS Statistics 23 software. One-way analysis of variance (for multiple comparisons) was performed on all metric data; for clinical scores and hind paw swelling, the area under the curve was calculated first. All categorical data (semi-quantitative scores) were analyzed by a Mann–Whitney U test.

## Data Availability

Data are available from the corresponding author upon reasonable request.
